# Sugar Lego: gene composition of bacterial carbohydrate metabolism genomic loci

**DOI:** 10.1186/s13062-017-0200-7

**Published:** 2017-11-25

**Authors:** Anna Kaznadzey, Pavel Shelyakin, Mikhail S. Gelfand

**Affiliations:** 1 A.A.Kharkevich Institute for Information Transmission Problems, RAS, Bolshoy Karetny per. 19, Moscow, 127051 Russia; 2 Vavilov Institute of General Genetics, Gubkin 3, Moscow, 119991 Russia; 3 Center for Data-Intensive Biomedicine and Biotechnology, Skolkovo Institute of Science and Technology, Moscow, 143028 Russia; 4 Faculty of Computer Science, Higher School of Economics, Kochnovsky pr. 3, Moscow, 125319 Russia; 5 Faculty of Bioengineering and Bioinformatics, M.V.Lomonosov Moscow State University, Vorobievy Gory 1-73, Moscow, 119991 Russia

**Keywords:** Comparative genomics, Carbohydrate metabolism, Bacterial genomics

## Abstract

**Background:**

Bacterial carbohydrate metabolism is extremely diverse, since carbohydrates serve as a major energy source and are involved in a variety of cellular processes. Bacterial genes belonging to same metabolic pathway are often co-localized in the chromosome, but it is not a strict rule. Gene co-localization in linked to co-evolution and co-regulation. This study focuses on a large-scale analysis of bacterial genomic loci related to the carbohydrate metabolism.

**Results:**

We demonstrate that only 53% of 148,000 studied genes from over six hundred bacterial genomes are co-localized in bacterial genomes with other carbohydrate metabolism genes, which points to a significant role of singleton genes. Co-localized genes form cassettes, ranging in size from two to fifteen genes. Two major factors influencing the cassette-forming tendency are gene function and bacterial phylogeny. We have obtained a comprehensive picture of co-localization preferences of genes for nineteen major carbohydrate metabolism functional classes, over two hundred gene orthologous clusters, and thirty bacterial classes, and characterized the cassette variety in size and content among different species, highlighting a significant role of short cassettes. The preference towards co-localization of carbohydrate metabolism genes varies between 40 and 76% for bacterial taxa. Analysis of frequently co-localized genes yielded forty-five significant pairwise links between genes belonging to different functional classes. The number of such links per class range from zero to eight, demonstrating varying preferences of respective genes towards a specific chromosomal neighborhood. Genes from eleven functional classes tend to co-localize with genes from the same class, indicating an important role of clustering of genes with similar functions. At that, in most cases such co-localization does not originate from local duplication events.

**Conclusions:**

Overall, we describe a complex web formed by evolutionary relationships of bacterial carbohydrate metabolism genes, manifested as co-localization patterns.

**Reviewers:**

This article was reviewed by Daria V. Dibrova (A.N. Belozersky Institute of Physico-Chemical Biology, Lomonosov Moscow State University, Moscow, Russia), nominated by Armen Mulkidjanian (University of Osnabrück, Germany), Igor Rogozin (NCBI, NLM, NIH, USA) and Yuri Wolf (NCBI, NLM, NIH, USA).

**Electronic supplementary material:**

The online version of this article (10.1186/s13062-017-0200-7) contains supplementary material, which is available to authorized users.

## Background

Bacterial carbohydrate metabolism is extremely diverse. Carbohydrates serve as a major energy source; they are also involved in a variety of cellular processes, such as the cell wall biosynthesis. This study focuses on a large-scale analysis of bacterial genomic loci related to the carbohydrate metabolism. It is a common knowledge that bacterial genes belonging to same metabolic pathway are often co-localized in the chromosome [1–5]. Numerous studies regarding gene functions are dedicated to specific operons within single genomes or genomes of closely related species. Comparative gene studies throughout the years influenced the understanding of reasons behind gene co-localization, revealing its importance in gene co-evolution and co-regulation and showing that physically interacting proteins tend to be encoded by genes co-localized in a specific order on the chromosomes [6]. Co-localization patterns could be shared fully or partially between species, not only due to common ancestry, but also to the horizontal transfer events [7, 8]*.* Chromosomal localization in combination with protein similarity is a useful tool for the prediction of gene function [9–13]*.*

We studied genes encoding carbohydrate transforming enzymes, such as hydrolases, phosphorylases, dehydratases, acetylases, etc., as well as related transporters and transcription regulators. We analyzed overall co-localization tendencies of these genes, belonging to a large and important segment of metabolism, simultaneously in a broad number of bacterial species.

Configurations that genes form in bacterial chromosomes, e.g. chromosomal gene clusters (without a reference to the operon structure, the order of genes, or their orientation) will be further called cassettes. In a recent study [14], 68.7% of 4.5 million protein-coding genes from prokaryotic genomes were found to form conserved cassettes based on their COG content (a cassette was considered conserved if the respective COGs (clusters of orthologous groups) [15] combination occurred at least twice among the studied genomes); thus about a third of genes seemed to behave as singletons and did not have apparent links to their genomic neighborhood. In well-studied genomes like *Escherichia coli* and *Bacillus subtilis,* about a third of known genes form monocystronic (single-gene) operons [16, 17]. One of our goals was to compare these results with cassettes composed solely of genes belonging to a specific segment of the metabolism, here, carbohydrate catabolism and synthesis. Around 148,000 genes and 264 different COGs were studied in 665 genomes of 30 bacterial classes. The studied genes had varying propensity towards being involved in cassettes or existing as singletons. We explored the influence of two major factors, gene functionality and species phylogeny, on these preferences, characterizing cassette-forming tendencies of each functional class and bacterial taxon. The variety of cassettes was assessed based on their size and gene content, which also allowed us to compare commonly found combinations with participants of known carbohydrate metabolic pathways. We further analyzed possible pairwise links between frequently co-localized genes from different functional classes and orthologous clusters. Finally, we studied cases of co-localized genes with similar functions, in particular, assessing the possibility of their origin by duplication. Altogether, we obtained a comprehensive picture on both global and local co-localization preferences of carbohydrate metabolism genes in bacteria.

## Methods

### Genomes and genes

The total number of analyzed genomes was 665, with a randomly selected single strain per specie (see Additional file 1). The total number of studied genes was approximately 148,000; the gene data were obtained from the IMG database [18], the majority of studied genes belonging to the “G” category, which contains annotation of genes associated with bacterial carbohydrate metabolism, including their known and predicted functions, locations on the chromosomes, and COG (cluster of orthologous groups) identification numbers (which are assigned in the IMG database to all genes by an automatic procedure, performing RPS-BLAST search for each gene against COG position-specific scoring matrices from the conserved domains database). Gene sequences were obtained from GenBank [19].

### Gene classification

A two-level classification system was developed based on the functionality and orthology clustering of genes. 273 COGs were initially selected from the IMG database “G” category, and 239 were found in the studied genomes after eliminating the strain bias as described above. Approximately 2% of genes had additional COG identification numbers, which often indicates gene fusion and serves as evidence of a functional relationship [20]. According to Mavromatis et al. [14], approximately 6% of all bacterial and archaeal genes are fusion products. Such cases in our study were further treated as co-localized genes. From the fusion data we selected 34 additional COGs; each of them contained genes suggested to be involved in the carbohydrate metabolism according to their annotations. Most of them belonged to “M” (“Cell wall/membrane/envelope biogenesis”), “R” (“General function prediction only” or “K” (“Transcription”) categories. An example of such case is COG4158 from the “R” category; the genes were annotated as "monosaccharide ABC transporter membrane protein, CUT2 family" and “ribose ABC transporter, permease protein”, hence, we assigned this COG to our “transporter” functional class. The final set comprised 264 COGs (Additional file 2).

### Cassette analysis

Cassettes were identified based on gene proximity in chromosomes. Genes were considered to form a cassette if they belonged to the previously described carbohydrate gene database and were located next to each other, with intergenic distances not exceeding 200 nt, as in the OperonDB project [21]. One 1500 nt gap was allowed per cassette, so roughly one additional gene not necessarily known to be involved in the carbohydrate metabolism or with an unknown function could not break a cassette. The order of genes in a cassette and their orientation were not taken into account.

All collected cassettes were analyzed based on their gene number, which will be further regarded as the cassette size, and sorted on both levels of the classification, by their COG content and by their functional content. The cassette diversity was studied by calculating the occurrence numbers for all cassette types. Abundant COG and functional patterns within cassettes were compared to known metabolic pathways obtained from Metacyc [22] and KEGG [23].

### Co-localization of functions

One of our goals was to study pairwise co-localization properties of genes belonging to different functional classes. To assess statistical significance of such links, we created a random model. We shuffled studied carbohydrate metabolism genes 10,000 times over their positions in each genome separately and calculated co-localization numbers for each pair of function classes, so that if each of the two function classes was present in a cassette at least once, it counted as a co-localization event. We used the obtained distribution to calculate the *p*-value for the observed co-localization events. If no such events occurred, the p-value of 1/10001 was assigned to the pair. The Bonferroni correction was applied for the number of analyzed pairs with the significance level (Alpha) 0.05. A similar analysis was carried out for the same-class gene co-localization, regarding cases when genes from the same functional class were present within the same cassette exactly two times, exactly three times, etc.

### Co-localization of COGs

We analyzed co-localization tendencies of different COGs within each pair of functional classes. To account for the COG size (the number of genes in a COG), we compared observed co-localization numbers for genes from different COGs with expected co-localization numbers, that depended only on COGs sizes, obtaining a chi-like value for each COG pair (squared difference between expected and observed values divided by the expected value). Co-localization numbers were clustered by the k-means algorithm implemented in Perl [24]. We iterated clustering with an increasing number of clusters and added penalty dependent on the squared number of clusters. The same procedure was carried out for the chi-square-like values. COG-pairs which belonged to clusters with highest values in both clusterizations were considered significantly abundant.

### Сomparison of gene sequences

To test whether same-COG genes within each cassette result from a duplication, we compared the sequences of such genes to each other and to all other genes of the same COG from our database, and searched for bi-directional best hits. We used the NSimScan tool [25] with the following parameters: -k 7 -t 80 --it 50 --xt 50 –mrep. NSimScan is a tool searching for similarities in nucleotide sequences. -k: is the “k-mer size”, regulating the word size in the lookup; −t: is the “k threshold”, specifying the diagonal score threshold that triggers further processing; −-it is the minimal percent identity at the minimal allowed alignment length; −-xt is the minimal percent identity at maximal possible alignment length; and --mrep parameter turns on the mode where only one best representative per group of repeating similarities is reported.

## Results and discussion

### Singleton genes and cassette variety

Fifty three percent of 148 thousand bacterial carbohydrate metabolism genes formed cassettes. This yields a significant role of singleton genes despite expectations of stronger clustering tendencies of functionally related genes based on recent literature [1, 4, 10, 14, 26, 27]. Several studies of evolutionary modules, however, suggest that genes from a number of well-studied bacterial metabolic pathways may not demonstrate conserved co-localization [3, 28], and our study extends this observation.

**Fig. 1:**
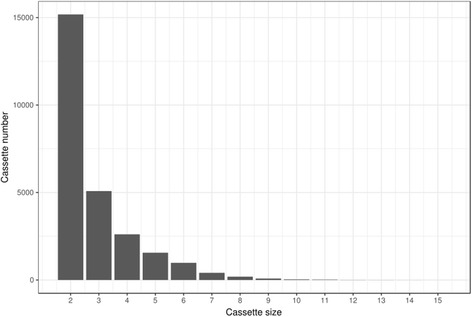
Gene cassette size distribution among 665 studied genomes

In total, the studied genes formed over 26 thousand cassettes. Most cassettes were short; 55% were two-gene, and 20% were three-gene cassettes (Fig. 1). The distribution of cassette sizes among different functional classes and different bacterial taxa is available in Additional file 3 and Additional file 4, respectively. Most distributions are similar to the general distribution in Fig. 1, with a few exceptions. An example are genes from the transporter functional class, which occur in 2-gene cassettes almost as often as they occur in 3-gene cassettes, most likely due to the abundance of large complexes such as ABC-systems, which consist of at least three subunits.

The cassettes comprised 10.4 thousand distinct COG combinations and 2.5 thousand distinct functional combinations. Based on the functional content, 45% of the cassettes were unique, occurring only once in the studied genomes. Only 43% of all studied genes coding for carbohydrate metabolism proteins belonged to conserved cassettes (a cassette was considered conserved if at least two cassettes with the same COG content were present in our database), in comparison to 69% of all protein-coding bacterial genes present within conserved combinations, according to Kyrpides et al. [14]. It seems that a large fraction of prokaryotic genes do not form evolutionarily conserved combinations, and for the carbohydrate metabolism genes this fraction is even larger. This effect can partly be explained by the possibility that some genes form evolutionarily s combinations with genes related to other segments of metabolism, for example, genes linked to nucleotide metabolism or other pathways which simultaneously involve carbohydrate residues linked with other types of molecules, such as glycolipids, glycoproteins, etc.

The fraction of genes from a given set located within cassettes was named as the cassette propensity of this set. Functional classes differed greatly in the cassette propensity, ranging between 23 and 93% (Table 1). Classes with the smallest cassette propensity were nucleosidases, phosphatases, and mutases (23, 38, and 42%, respectively). Again, one of the explanations for such low cassette propensity could be involvement of respective proteins in other types of metabolism. For instance, nucleosidases produce monosaccharides and hence belong to the carbohydrate metabolism, but they are also linked to nucleotide metabolism pathways, and may form s combinations with genes from the latter.

**Table 1:** Functional classes of genes

Functional class	Enzyme EC number	Number of genes	Cassette propensity
Transcriptional	Not applicable	39,136	35,29%
Transport	Not applicable	29,701	70,83%
Glycosyltransferase	2.4.1.	14,579	62,30%
Glycosidase	3.2.1.	11,475	64,74%
Kinase	2.7.1.; 2.7.9	9250	57,95%
Isomerase	5.3.1.	6458	55,20%
Dehydrogenase-OH	1.1.	5518	57,67%
Decarboxylase	4.1.	2788	58,97%
Nucleotidyltransferase	2.7.7.; 2.7.8	2125	70,96%
Dehydratase	4.2.	2091	52,75%
Phosphatase	3.1.3.	2036	37,77%
Epimerase	5.1.3.	1753	61,78%
Deacetylase	3.5.1.	1525	51,02%
Transaldolase/transketolase	2.2.1.	1514	70,54%
Mutase	5.4.2.	1502	40,35%
Carboxylic-esterase	3.1.1.	1153	63,49%
Dehydrogenase-O	1.2.	781	69,78%
Nucleosidase	3.2.2.	597	23,28%
Malto-oligosyltrehalose synthase	5.4.99	100	93,00%

Functional classes of carbohydrate metabolism genes (assigned according to the Enzyme Nomenclature classification obtained from the IMG database [14]), number of genes in each class, and their tendency towards localization within carbohydrate metabolism cassettes (cassette propensity)

The highest propensity of 93% was demonstrated by the small class of malto-oligosyltrehalose synthases, followed by the transaldolases/transketolases and, understandably, by the transporter class. The latter is due to the fact that, as mentioned above, many transporters, such as the ABC or the PTS systems, consist of multiple subunits encoded by genes that are often organized in operons.

**Fig. 2:**
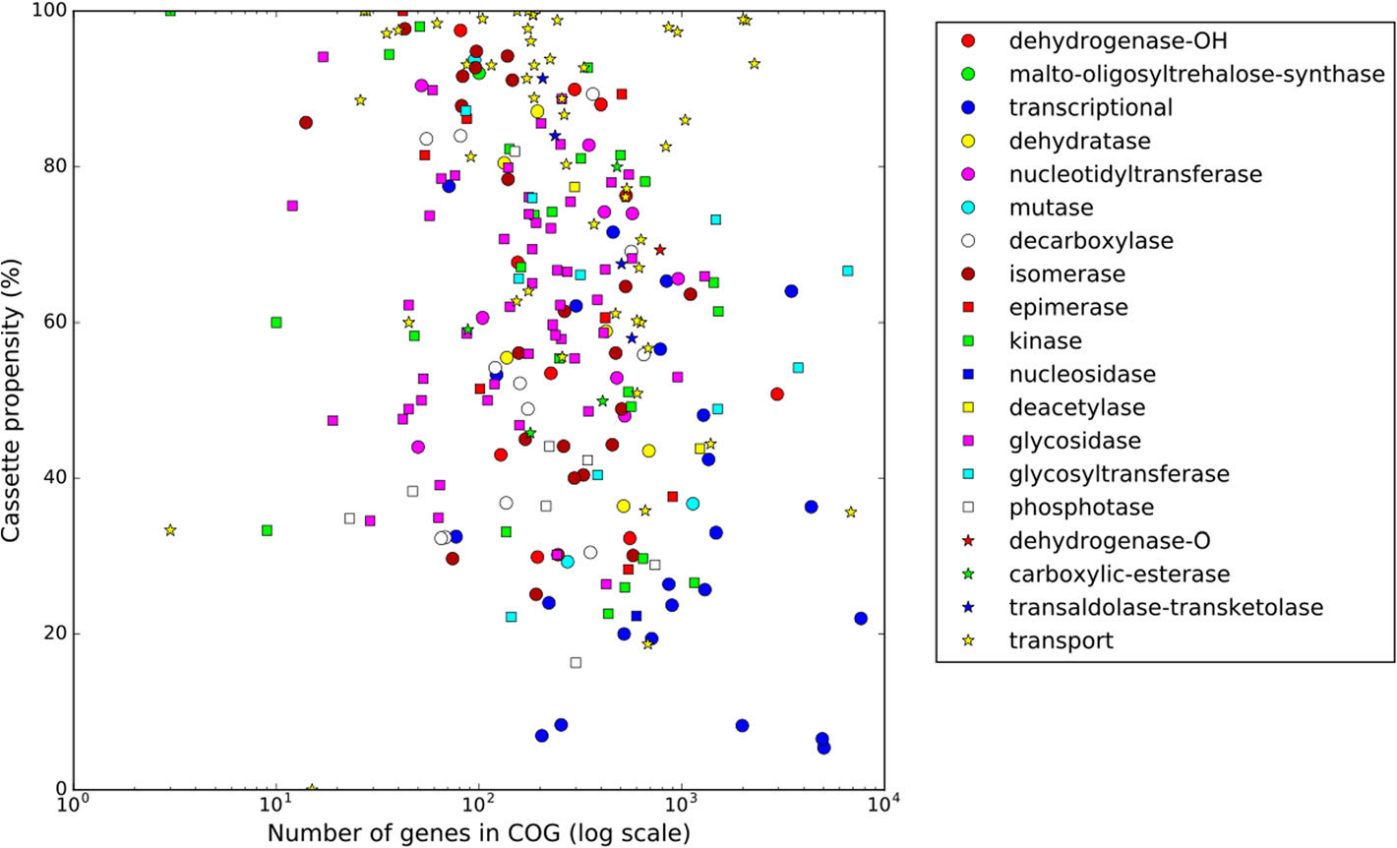
Cassette propensity of genes from different COGs. Genes from different COGs within the same functional class may have different cassette propensity. Dots represent different COGs, shape and color of each dot indicates its functional class, stated on the right

The cassette propensity for different COGs varied even stronger, ranging from 0 to 100% (Fig. 2). Large COGs (containing over four thousand genes) had a significant fraction of singletons; the cassette propensity for most of them, including secondary transporters of the MFS superfamily and transcriptional regulators, was less than 40%. The exception is glycosyltransferase COG0438, comprising 6587 genes, which is involved in the cell envelope biosynthesis and has cassette propensity of 66%. Some medium-sized COGs (two to four thousand genes), on the other hand, had the cassette propensity of over 90% (for example, the ABC-transporters mentioned above). The smallest COGs (less than two thousand genes) with a high propensity belonged to the dehydrogenase, isomerase, kinase, epimerase, and transaldolase/transketolase classes. The propensity distribution for COGs from different functional classes is available as a histogram in Additional file 5.

**Fig. 3:**
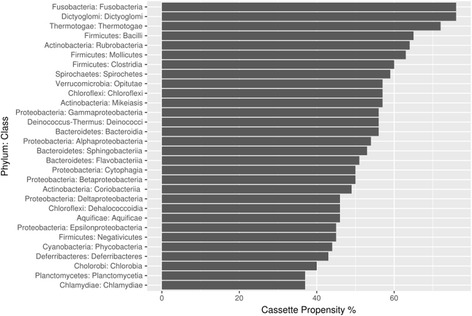
Cassette propensity among bacterial taxa. Cassette propensity of carbohydrate metabolism genes for different phyla and classes of bacteria

The phylogeny played an important role in the cassette propensity; for different bacterial taxa it varied between 37 and 76% (the analysis was restricted to taxa with at least two genomes with at least a hundred annotated carbohydrate metabolism genes in each) (Fig. 3). Taxa with the highest cassette propensity were Dictyoglomales and Fusobacteriales (76%), Thermotogales (72%), and Lactobacillales (65%), consistent with known preference of some of these bacteria (e.g. the *Streptococcus* species [16]) towards long operons. The bacteria with the smallest cassette propensity were Planctomycetia (37%), Chlamydiae (37%), Chlorobia (40%), Deferribacteres (42%) and Cyanobacteria (43%). Among large taxa, with over eight thousand annotated carbohydrate metabolism genes in each, Deltaproteobacteria had the lowest cassette propensity (46%), Betaproteobacteria were in the middle with 50%, Alphaproteobacteria, Gammaproteobacteria and Actinobacteria had the cassette propensity slightly above average (54, 56, and 57%, respectively), while Clostridia and Bacillales leaned towards higher values (60 and 64%, respectively).

The most common protein functions encoded by genes involved in cassettes were transporter, glycosidase, and glycosyltransferase. The longest cassette (found in *Stackebrandtia nassauensis* DSM 44728), comprising fifteen genes, contained eleven transporters, two isomerases, one glycosidase, and one glycosyltransferase. Transporter genes occurred in 18% of all cassettes; 10% cassettes contained two or more. Glycosidases were found in 19% of all cassettes; 5.8% cassettes had at least two glycosidases, and 1.7% had at least three (the highest number was seven glycosidases in a cassette, in *Prevotella ruminicola* 23 and *Bifidobacterium dentium* Bd1). Glycosyltransferases were found in 19% of all cassettes; 9.4% cassettes had at least two glycosyltransferases, and 3.3% had at least three (the highest number was nine glycosyltransferases in a cassette, in *Pedobacter saltans* DSM 12145 and *Bacillus weihenstephanensis* KBAB4). No functional class was present in more than a fifth of all studied cassettes, which points to a significant diversity of gene co-localization patterns linked with the bacterial carbohydrate metabolism.

### Co-localization of genes from different functional classes

**Fig. 4:**
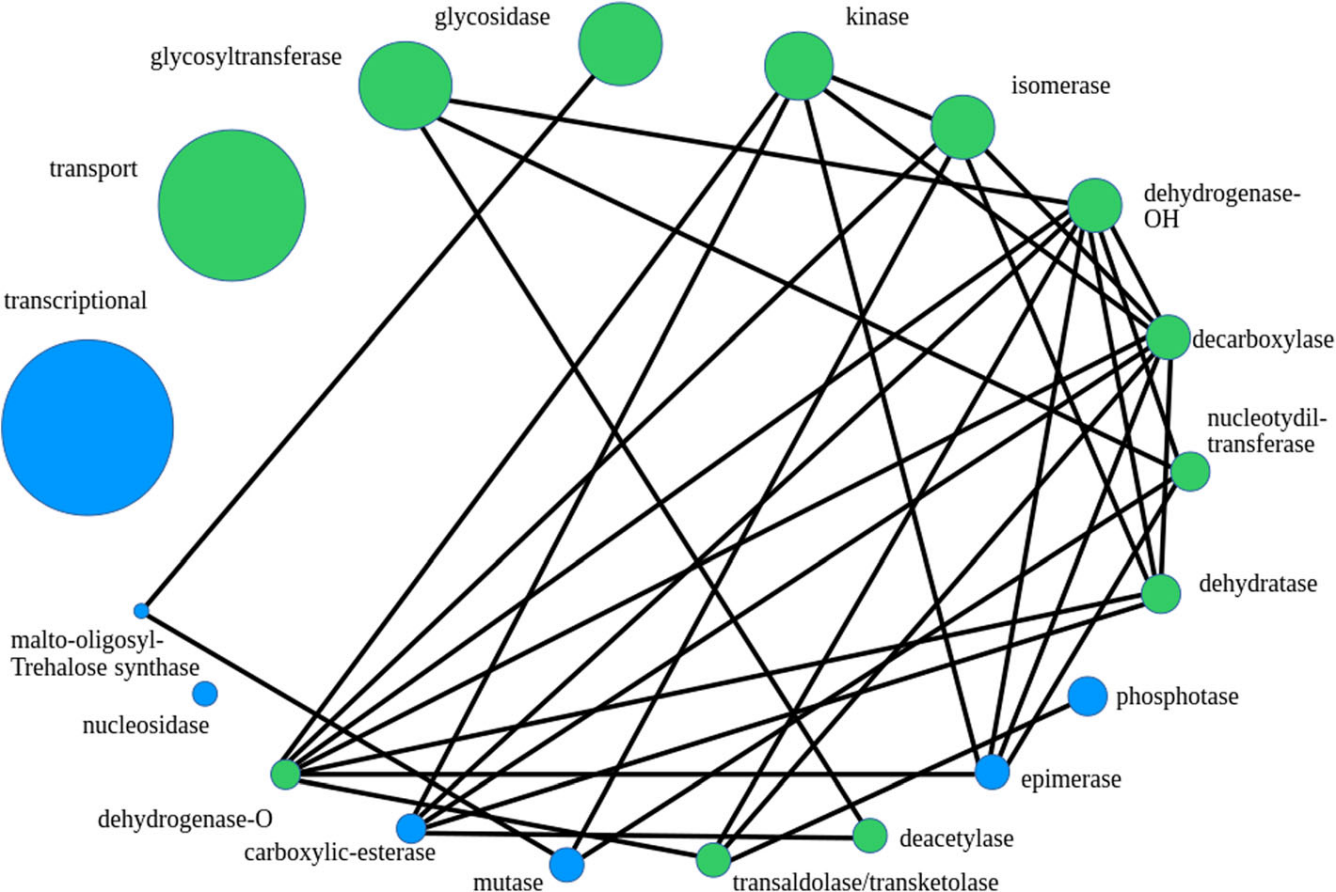
Links between functional classes. Bubbles represent functional classes. The size of a bubble corresponds to the relative class size. Black lines connect classes which form the co-localization links. Green color of a bubble indicates that genes from the respective class have a significant tendency to co-localize with each other

To explore patterns of the cassette composition, we compared co-localization counts of genes of various functions within real cassettes and in random simulations as described in the Methods section; obtained counts are listed in the Additional file 6. Out of 190 possible pairs of functions for the nineteen studied classes, 45 had a gene co-localization count higher than random (with a calculated *p*-value of 0.0001 or less), indicating the presence of a functional link (Fig. 4). Only 24% of all possible pair links had passed the given criteria, despite expectations for many functional classes to show distinct co-localization preferences reflecting abundant metabolic reaction adjacencies.

The number of links per class varied between zero and eight. The overall abundance of genes with a certain function did not necessarily yield a large number of links involving this function; for example, the transporter genes, despite being a large class with over twenty-one thousand genes located within cassettes, did not form any links. The size of a class, overall, did not determine the number of links; for example, the transaldolase/transketolase class with only a thousand genes located within cassettes formed six links, while the similarly sized deacetylase class formed only three. The cassette propensity of a class did not necessarily determine the number of its links, either. The decarboxylase class with the cassette propensity of around 60% was involved in eight links (the maximum observed number of links per class), while the glycosyltransferase class with a similar cassette propensity formed only four. Most of the 45 links were formed by genes from the decarboxylase, dehydrogenase-OH, and dehydrogenase-O classes (having eight, eight, and seven links, respectively), meaning that these classes have the most diverse, yet non-random preferences towards the neighbor functions.

Most of the significant links consisted of functions forming known and abundant metabolic pathways. The isomerase-kinase pair is present in many pathways including lactose, galactose, chitin, and arabinose degradation; the decarboxylase-kinase pair is present in all variants of the Entner-Doudoroff pathway; the epimerase-mutase pair is found in the glycolysis/gluconeogenesis-related pathways and the mannan degradation pathways; the dehydrogenase-carboxylic esterase pair is involved in the galactose degradation pathways [21, 23]*.* This is consistent with observed tendencies in many specific cases, of proteins that are parts of same metabolic pathways to be encoded as genes co-located on the chromosomes within same genomic loci or operon [3, 4, 6]. However, many pairs of functions, present in many known metabolic pathways, such as the glycosidase-kinase pair or the glycosyltransferase-kinase pair, did not pass the significance criteria; this yet again points to the fact that gene co-localization is not a strict requirement for consequent enzyme functions.

Pairwise combinations of functions are formed by pairwise combinations of COGs. We analyzed the most abundant COG pairs for each pair of classes. Each class pair contained up to ten such pairs; up to two of them had also passed the statistical significance filter, described in the Methods section. These results are listed in Additional file 7.

Linked functional pairs sometimes formed apparent three-way connections. For some of them, e.g. for the kinase, isomerase, and dehydrogenase-O classes, the respective three most abundant COG pairs for three of the possible class pairs were comprised of three COGs in total, so all these COGs were compatible with each other, and the respective three-COG combination also existed in a number of genomes. However, this was not a universal case, e.g. for pairs formed by the nucleotidyltransferase, glycosyltransferase, and dehydrogenase-OH classes, the respective three most abundant COG pairs were formed by six different COGs, whereas their cross-combinations were rare.

We suggest that a combined study of functional and COG links, and, in particular, three-way connections, may provide new data for COG annotation. For instance, the most abundant nucleotidyltransferase-glycosyltransferase pair consisted of COG0448, annotated as glucose-1-phosphate adenylyltransferase (EC 2.7.7.27), and COG0297, annotated as glycogen synthase (EC:2.4.1.21), that uses ADP-glucose; and indeed, these two enzymes are involved in two consequent steps of starch metabolism pathways. Similarly, the most abundant nucleotidyltransferase – dehydrogenase-OH pair consisted of COG1091, annotated as dTDP-4-dehydrorhamnose reductase (1.1.1.133), and COG1209, annotated as glucose-1-phosphate thymidylyltransferase (EC 2.7.7.24); both these enzymes are part of the dTDP-6-deoxyhexose biosynthesis pathway. On the other hand, the annotations for both members of the most abundant COG pair formed with the glycosyltransferase and dehydrogenase-OH classes (COG0451 and COG0438) are not as clear. Both are large gene clusters, over six thousand and over thirteen thousand genes, respectively, and contain genes encoding proteins of many various predicted functions such as dTDP-4-dehydrorhamnose reductase for COG0451 and glycogen synthase for COG0438. The respective genes are co-localized over a thousand times in various bacterial taxa, more often than any other dehydrogenase-OH and glycosyltransferase COG pair. Such distinct co-localization may indicate a strong functional and evolutionary relationship between these genes, and is a reason to further investigate the functional specifics of respective proteins and their biological roles.

### Co-localization of genes with similar functions

Twelve out of forty-five of the observed functional links were formed by same-class pairs, which meant that twelve out of nineteen functional classes demonstrated a significant tendency for co-localization with genes from the same class (Table 2).

**Table 2:** Co-localization of genes from same functional classes

Functional class	Genes in cassette	Number of cassettes	*P*-value
Transaldolase/transketolase	2	280	< E-300
	3	6	2,29E-042
	4	2	< E-300
Kinase	2	619	2,28E-025
Dehydratase	2	61	1,51E-019
Nucleotidyltransferase	2	101	1,96E-084
Isomerase	2	375	1,53E-033
	3	26	4,55E-007
Decarboxylase	2	112	9,69E-029
	3	3	6,50E-002
Dehydrogenase-OH	2	335	2,61E-075
	3	33	4,94E-037
	4	3	2,24E-007
Deacetylase	2	34	1,47E-009
Dehydrogenase-O	2	16	6,02E-031
Glycosyltransferase	2	1593	2,62E-276
	3	578	< E-300
	4	195	< E-300
	5	58	< E-300
	6	18	< E-300
	7	11	< E-300
	8	3	< E-300
	9	2	< E-300
Glycosidase	2	1094	1,99E-063
	3	321	5,02E-161
	4	87	1,96E-121
	5	24	1,86E-099
	6	5	7,12E-042
	7	2	1,29E-008
Transport	3	2648	< E-300
	4	931	< E-300
	5	157	7,99E-037
	6	59	6,51E-029
	7	18	7,42E-014
	8	5	3,85E-005

Genes from twelve out of nineteen classes demonstrate a tendency towards localization with genes from the same class. For each number of class representatives in a cassette, the number of such cassettes among studied genomes is given. The P-value indicates probability of obtaining these co-localization numbers in a random distribution

Classes with the largest fraction of co-localized same-class genes were transporter, glycosidase, transketolase/transaldolase, and glycosyltransferase (Table 3).

**Table 3:** Genes localized within cassettes with same-class neighbors

Function	Genes with same-class neighbors
Transport	78,10%
Glycosyltransferase	68,45%
Transaldolase-transketolase	54,87%
Glycosidase	49,31%
Transcriptional	40,71%
Kinase	25,88%
Dehydrogenase-OH	24,54%
Isomerase	23,34%
Decarboxylase	14,42%
Nucleotidyltransferase	13,40%
Dehydratase	11,33%
Deacetylase	9,13%
Dehydrogenase-O	5,87%
Nucleosidase	4,32%
Carboxylic-esterase	3,01%
Mutase	2,97%
Epimerase	2,77%
Malto-oligosyltrehalose synthase	2,15%
Phosphatase	0,00%

Percentage of genes from each functional class localized within carbohydrate metabolism cassettes (not singletons) with other same-class genes also present within cassettes

Interestingly, glycosyltransferases and transaldolases/transketolases were more often found several times within a cassette than as a single representative of a given function. Genes of the same class co-localized within cassettes can be divided into two groups: genes encoding subunits of protein complexes, and genes encoding separate proteins. The most common example of the former are represented by the transporter functional links with at least three transporter genes involved. Cassettes with only two transporter genes did not pass the threshold for the Bonferroni correction; this can be explained by the common multi-domain structure of transporter complexes, such as ABC-transporters, which require three genes encoding three respective subunits. Same-class gene pairs from most other classes encode independent proteins, which, in some cases, may be involved in the same metabolic pathway. For instance, several glycosidases may be required for different stages of polysaccharide degradation, and studies show that the respective genes may belong to a single operon or several co-localized operons; e.g., in *Gramella forsetii* the laminarin utilization operon contains three glycosidases, and two adjacent alpha-1,4-glucan utilization operons contain four glycosidases [29]. Several glycosyltransferases are often involved in the cell wall biosynthesis; indeed, genomes of *Lactococcus lactis* and other lactic acid bacteria may contain more than seven glycosyltransferase genes per operon [30]. Transaldolase and transketolase enzymes are involved in the pentose phosphate pathway, and the respective genes, such as *E. coli talA* and *tktB* genes, may be co-localized. As for less frequent cases, it is known, for example, that two or three kinases may be involved in different phosphorylation processes within the same pathway, such as the lactose degradation [22, 23]; and it would explain co-localization of the respective genes. On the other hand, for many same-class gene pairs, such as the decarboxylase-decarboxylase pair, the reason for co-localization is not obvious.

One of the mechanisms behind co-localization of genes with similar functions is local duplication. It is likely if genes from a pair also belong to the same COG, which was 44% of the cases, involving 189 out of 264 studied COGs. We calculated the alignment score for genes from each of such pairs using the NSimScan tool as described in the Methods section. We then found the best matches for both genes among other genes from all studied genomes. These scores were compared. Only in 3.6% cases same-COG genes created a bi-directional best hit with each other. In all other cases the genes had better matches located elsewhere, and in 62% these better matches were not located within same cassettes or even same genomes.

Paralogs are subject to significantly weaker purifying selection than orthologs [31], so these results do not rule out the duplication origin for the co-localized genes which have the same best match with a single third gene. In this scenario the original pair could be a result of a local duplication, where each gene from a pair has been evolving faster compared to its non-duplicated orthologs in other genomes, thus they are both more similar with these orthologs than they are with each other. However, over 90% of same-COG pairs had two different best matches for their genes.

Overall, it seems that the vast majority of co-localized genes of the same class did not originate from recent local duplications. An explanation behind co-localization of same-COG genes could also be xenologous gene displacement, where one of the ancestral genes from a pair of duplicated genes is replaced via horizontal transfer with a gene from an outside source. Such pseudoorthologs acquired from different sources are called xenologs. Another reason could be acquisition of pseudoparalogous genes, where a homologous gene is transferred next to the original gene from another genome without replacements [32].

Genes of similar function located together on the chromosome, especially in very large clusters like glycosyltransferases and glycosidases, could perhaps work as a ‘screwdriver set’, being expressed when a number of similar actions are required simultaneously, so respective proteins would perform similar tasks in the carbohydrate degradation or biosynthesis processes. Simultaneous expression of genes with similar functions could be important, for example, during the degradation or biosynthesis of complex polysaccharides, or in poor environmental conditions requiring utilization of all possible carbohydrates. It is known that under glucose starvation some bacteria, for instance, the *Bacillus* species, are capable of simultaneous activation of many genes responsible for the catabolism and transport of alternative carbon sources [33, 34]; this concerns, in particular, many transporter and hydrolase genes; it might be that co-localization of genes with similar functions is useful for the regulation of transcription in such cases.

## Conclusions

We present the results of a thorough large-scale exploration of the chromosomal organization of genomic loci related to the bacterial carbohydrate metabolism. Evolutionary relationships of genes, manifested as co-localization patterns, differ between gene functions, orthologous gene clusters, and bacterial taxa. Overall they form a complex and diverse system with a significant role of singleton genes and very short gene cassettes. In 665 bacterial genomes only 53% of 148 thousand studied genes were co-localized with other carbohydrate metabolism genes, and 55% of the cassettes they formed contained only two genes.

Two major factors influencing the carbohydrate metabolism gene tendency to co-localize (the cassette propensity) were found to be gene function and bacterial phylogeny. The cassette propensity varies between 23 and 93% in different gene functional classes, with malto-olygosyltrehalose synthase, transporter, and transaldolase and transketolase classes having the highest cassette propensity. It varies between 40 and 76% among bacterial taxa, with the highest cassette propensity observed in Fusobacteriales, Dictyoglomales, and Thermotogales classes.

We demonstrated forty-five significant pairwise co-localization links between functional classes of genes. The number of such links varied from zero to eight per class. Decarboxylase and dehydrogenase genes showed the most diverse and specific preferences towards functions of neighboring genes, while transporter genes and glycosidase genes, despite being involved in a large number of cassettes, did not show any significant preferences. Different COGs were involved in the links between classes. Characterization of the most abundant COG pairs for each class pair may be a source for hypothesis about specific functions of the involved genes with subsequent experiment analysis.

Genes from eleven functional classes also demonstrated tendency to be co-localized with genes from the same functional class. The respective proteins could either be parts of complex systems, such as bacterial transport system, or work separately, in such processes as polysaccharide degradation or cell wall biosynthesis, where several glycosidases or glycosyltransferases are involved simultaneously. Genes from transporter, glycosidase, and glycosyltransferase classes are most common among same-class co-localization events, and genes from the transketolase and transaldolase and the glycosyltransferase classes are more often found in cassettes multiple times than as single representatives. Most of these same-class co-localization do not seem to originate from recent local duplications. Our study thus highlights the previously undescribed large-scale evolutionary tendency towards co-localization of genes with similar functions.

## Reviewers’ comments

### Reviewers’ report 1

**Dr. Daria V. Dibrova, A.N. Belozersky Institute of Physico-Chemical Biology, Lomonosov Moscow State University, Moscow, Russia (nominated by Armen Mulkidjanian, University of Osnabrück, Germany)**

### Reviewer comments

The manuscript "Sugar Lego: Gene composition of bacterial carbohydrate metabolism genomic loci" by A. Kaznadzey, P. Shelyakin and M.S. Gelfand describes a large-scale analysis of gene co-localization involving a COG-based (after Cluster of Orthologous Groups) classification of genes. The gene co-localization is one of important methods for uncovering protein functions and assigning correct genome region annotation, however many key papers on this subject were done more than a decade ago, and the field could now be revisited in much broader context. In sum, I find the topic of this paper actual and promising. The manuscript can be published after a minor revision.

Author’s response: *We thank the reviewer for the comments that allowed us to improve the manuscript.*

1. The reader would be very interested in a complete list of COGs that were analysed, with their names and functional classification according to the COG database, sorted into functional classes mentioned in the paper. If supplied together with the results, such information as e.g. cassette propensity values for each COG, number of members attributed to a COG and its “best neighbors” on cassettes, would serve as a large-scale result summary and a material for further studies (as suggested for the cases of “three-way connections”, line 285).

Author’s response: *We have created a (*Additional file 2 *) with detailed information on each studied COG, including all parameters suggested in this comment (NCBI COG description, gene number, cassette propensity,* etc.*). We also provide the propensity percentile data based on the overall COG propensity distribution. Instead of “best neighbors”, obtained through a filtering procedure based on comparison with random distributions (the data is already available in the existing* Additional file 7 *, previously called* Additional file 3 *), here we provide a list of the most common neighbors from the three most frequent cassettes for each COG.*

2. Those COGs that showed tendency to group with themselves or other COGs from the same functional class could be additionally marked. Currently they are not listed in the Additional files (squares in the main diagonal of Additional file 3 are empty, whereas Table 3 and the text indicate multiplicity of such cases).

Author’s response: *We have added the contents of the main diagonal in* Additional file 7 *(previously* Additional file 3 *), which include the data on frequently co-localized COGs from same classes.*

3. It is well-known that large amount of bacterial genes does not belong to any COG. However, the procedure used by the authors should allow such genes to enter cassettes. Information on the “COG-less” genes within cassettes related to the carbohydrate metabolism set might be potentially very interesting, so that such cases should be described and discussed.

Author’s response: *In this study we have used carbohydrate metabolism genes from the IMG database. Each of these genes has at least one COG assigned to it as a result of an automatic procedure, description of which we have added to the* *Methods* *section. Thus we do not have any “COG-less” genes in our analysis. Some poorly annotated genes not described in the IMG database could be present in our cassettes in the “invisible mode”, because one 1500 nt gap is allowed per cassette (which is approximately the length of one extra bacterial gene and its intergenic regions). Our procedure did not include identifying such genes, which were either absent from the IMG database, or were not annotated there as carbohydrate metabolism related genes. It could be an interesting subject for annotation based on chromosome co-localization, but it is out of scope of this study.*

4. A vector format would be better for Figs. 1, 2 and 3. Perhaps their current low resolution is an artifact of the manuscript packing for a review.

Author’s response: *We are providing images in a better resolution. Also, during the review process we have identified an error in* Fig. 2 *(demonstrating cassette propensity of COGs from different functional classes), caused by an incorrect scaling procedure (mentioned in further comments). We have updated the figure and the respective commentary. We have also created an addition to* Fig. 2 *in the form of a histogram which shows the distribution of different functional classes over cassette propensity (*Additional file 5 *).*

5. Line 62: the COG abbreviation is used without being introduced, and respective papers (Tatusov et al., 1997, or the most recent Galperin et al., 2015) are not cited in the manuscript. Further (lines 69–70) the following expression is used: “orthologous clusters of genes (COGs)”. The expression appear to be misleading, because: 1) it appears that all clusters of genes which are mentioned are orthologous and 2) even if this expression would be rephrased as “clusters of orthologous genes” (as in line 89), a single COG is not a cluster of orthologous genes, but rather a cluster of orthologous groups of genes, i.e. not all genes inside a COG are true orthologs. This is particularly important, because the authors use an assignment of proteins to COGs from an external database; the assignment appear to be based on sequence similarity to COGs, but not on a direct de novo COG construction.

Author’s response: *As explained in response to Comment 3, here we have used annotations provided by the IMG database, where genes are assigned to COGs by comparing protein sequences to COG PSSMs from the CDD database using RPS-BLAST. It is not a* de novo *construction, and COG numbers here indeed represent groups of orthologous clusters of genes and are provided by an outside source. We have added an explanation to the* *Methods* *section. The article on the NCBI COG database is now cited.*

6. Line 85: "The total number of analyzed genomes was 665, with a randomly selected single strain per specie" - from which initial sample were these 665 genomes sampled? Additional file 1 would benefit from adding taxonomic information, which will, for instance, reveal the domination of proteobacteria (291 species from 665 are proteobacterial).

Author’s response: *The selected species have been taken from the set of the IMG database bacteria species with annotated carbohydrate metabolism genes. We have added the taxonomic information on each species to* Additional file 1 *.*

7. Line 86: "The total number of studied genes was 148 thousand" Better to use numbers. Also it relates to the line 69, as well as other cases where numbers are written as text.

Author’s response: *We have switched words to numbers, as suggested.*

8. Line 87: It could be important to mention whether this “G” category in the IMG is the functional classification of original COGs, or an IMG classification.

Author’s response: *The “G” category (“Carbohydrate transport and metabolism”) is an original category from the NCBI COG database. We have added this explanation to the* *Methods* *section.*

9. Lines 96–97: Did I understood correctly that the cases of multi-COG proteins "were further treated as co-localized genes"? How many multi-COG proteins were in your sample? How did this affect the shuffling procedure (were they shuffled independently or not)?

Author’s response: *Multi-COG proteins were treated as co-localized genes. We have added respective commentary about their numbers to the manuscript. During the shuffling procedure they were shuffled independently.*

*We have analyzed in depth a number of individual cases and could not devise an explicit procedure for the separation of domain fusions and multiple predicted functions for a single, non-fused gene. At that, this is an important issue for the genome annotation in general, addressed, in particular, by Tripp* et al. *(Nucleic Acids Research, 2011) which has yielded global reannotation in GenBank and resolution of most of such ambiguities. Developing a new procedure which would allow us to resolve the remaining cases in our dataset was out of scope of this study, but in any case potential fusions should not have affected the results, since they have been observed in only 2% of the genes.*

10. Lines 97–99: It would be nice to see additional information on COGs which were added to the analysis being identified as fused with known COGs of carbohydrate metabolism within genes. The reason that "each of them contained at least several genes suggested to be involved in the carbohydrate metabolism according to their annotations" appear to be blurry; how are these COGs named and classified in the COG database itself?

Author’s response: *We have added a more detailed description on the process of adding new COGs to the database; we have also corrected the information on the total number of COGs obtained from the fusion study. Fusion-derived COGs are now marked green in the* Additional file 2 *, where their general NCBI descriptions are also available. We have also added an example of genes annotation of such COG from the IMG database, which revealed its link with carbohydrate metabolism and allowed us to include it in our study.*

11. Line 121–122: From my point of view, the paper would win greatly if the results of the performed comparison with established pathways would be added to the manuscript, perhaps as a scheme for most interesting cases. The functional classification used in the paper is too general to connect it with biological realm. Could at least the most interesting interactions between functional classes and COGs be mapped on a reaction scheme?

Author’s response: *Here, we aimed at finding general co-localization connections between major gene functions and COGs. These results are potentially applicable in pathways analyses, but the latter are out of the scope of this study. However, we provide a number of examples in the section "* *Co-localization of genes from different functional classes* *" and "* *Co-localization of genes with similar functions* *", where we compare obtained functional links and COG co-localization tendencies with several known metabolic pathways and reactions.*

12. Lines 127–128: The explanation of the shuffling procedure could be expanded. Which “studied genes” were shuffled - only those of carbohydrate metabolism or all genes?

Author’s response: *Here, we studied only carbohydrate metabolism genes, and to create a random model, we shuffled all 148 thousand of them over their positions (both cassette genes and singletons). We have added respective comment to the* *Methods* *section.*

13. Line 151–152: A short explanation of what does the NSimScan tool does and the meaning of parameters used is required here, so that the reader can understand what extent of similarity between the sequences compared to other proteins in COG is sufficient to claim duplication. It should be also explained what does “duplication” here means; the explanation from lines 358–359 could be moved here.

Author’s response: *NSimScan is a published tool searching for similarities in nucleotide sequences. We have added its description to the respective* *Methods* *section, along with description of the parameters. We have also moved the description of the duplication search criteria, as suggested.*

14. Line 105: typo in Table 1, “phosphotase” should be “phosphatase”. It is also repeated in Additional file 3. Also “malto-oligosyltrehalose” appear to spell different, as “maltooligosyl trehalose”.

15. Figure 2: the X axis is labeled log (Number of genes), while values on it are given as 103, 104 etc. It should be 3, 4 etc.?

Author’s response: *Corrected with thanks.*

16. Figure 3: What is “Proteobacteria: Cytophagia” (12th bar)? To my knowledge, the parent taxon for Cytophagia class is Bacteroidetes.

Author’s response: *Cytophagia class indeed belongs to Bacteroidetes, corrected.*

### Reviewers’ report 2

**Igor Rogozin, NCBI, NLM, NIH, USA**

### Reviewer comments

This study focuses on a large-scale analysis of bacterial genomic loci related to the carbohydrate metabolism. The authors described the complex system of evolutionary related genomic neighborhoods of bacterial carbohydrate metabolism genes.

Author’s response: *We thank the reviewer for the comments that allowed us to improve the manuscript.*

I do not see major methodological problems. I have questions about the “Cassette analysis”: "Cassettes were identified based on gene proximity in chromosomes. Genes were considered to form a cassette if they belonged to the previously described carbohydrate gene database and were located next to each other, with intergenic distances not exceeding 200 nt. One 1500 nt gap was allowed per cassette..." I think that these conditions (200/1500) are more strict than the usual analysis of gene pairs (that requires only co-localization). These thresholds look reasonable and may be an ad hoc result of the previous experience. I am sure these thresholds improved the accuracy of prediction and final quality of functional inferences. Did the authors try to optimize those parameters? Could variation of these parameters improve quality of neighborhood prediction in future studies or the authors are confident that these are (nearly-)optimal parameters? Any comments will be helpful for other researchers in the field.

Author’s response: *The threshold for gene co-localization on the chromosome was selected based on the literature, where 200 nt is considered an adequate maximum distance between bacterial gene neighbors. For instance, it has been used in gene pair analysis in the OperonDB in (Ermolaeva* et al.*), and we have added this reference to the* *Methods* *section. In some cases, even stricter parameters have been used,* e.g.*, 50 nt in (Salgado* et al.*) for operons in Escherichia coli. The 1500 nt gap is roughly a length of one bacterial gene with two intergenic distances.*

*In a preliminary study, we allowed for a 15 k nt distance (assuming that longest cassettes will be about 12–13 genes). This, however, led to inaccuracies, as multiple cassettes could be grouped into one, while in fact they were intersperced with multiple non-carbohydrate metabolism genes.*

Minor issues:

I think that this may be a typo: "we describe the complex system formed by evolutionary relationships of bacterial carbohydrate metabolism genes, manifested as co-localization patterns". I am not sure that the “system” may be formed by “relationships” in this context. I think that this sentence require some modifications. I am not sure.

Author’s response: *Changed “system” to “web”.*

### Reviewers’ report 3

**Yuri Wolf, NCBI, NLM, NIH, USA**

### Reviewer comments

Kaznadzey and co-authors survey genes related to carbohydrate metabolism in a wide selection of bacterial genomes, with the specific focus on gene co-localization. They find that, like most other genes in bacterial genomes, sugar-related genes form cassettes ranging in size from trivial (singletons) up to 15 genes long. The authors also find that the propensity to form non-trivial cassettes depends on the gene class (EC number) and the host taxonomy. Both the approach used by the authors and the results are quite reasonable and provide a potentially useful quantitative benchmark for further studies of links between the function and evolution in microbial genomes.

Author’s response: *We thank the reviewer for the comments that allowed us to improve the manuscript.*

In my experience the simple number (or fraction) of co-localized genes is a useful, but somewhat information-poor measure of the degree of co-localization. I would be tempted to quantify the co-localization as a property of the distribution of the cassette size, starting with the overall distribution presented in Fig. 1 (is it better approximated as an exponent? a power law? something else?), and proceeding to changes in its shape or parameters for subsets of the data (gene classes and bacterial taxa). This has a chance to provide a better resolution.

Author’s response: *For a more detailed analysis of the cassette size distribution analysis, we calculated the cassette size distribution for different functional classes and bacterial phyla. Most distributions are similar to the general distribution in* Fig. 1 *, with several exceptions. One of them is the transporter functional class, which occurs almost as often in 2-gene cassettes as it does in 3-gene cassettes, due to large transporter complexes, such as ABC-transporters; the other is malto-oligosyltrehalose-synthase class, which is the smallest functional class (containing only 100 genes), which most likely does not yield a s distribution. We have added respective comment to the Results section.*

*Also, in order to improve resolution on other sets of data, we have constructed a histogram showing the distribution of COGs from different functional classes over cassette propensity (*Additional file 5 *), and now provide percentile information regarding position in the overall COG propensity distribution for each COG in the* Additional file 2 *.*

The lack of significant co-localization links between the transcriptional regulation class and the rest of the carbohydrate metabolism genes requires some attention. Is the identification of transcriptional regulators as the members of carbohydrate metabolism cassettes specific enough? Is the regulation occurs mostly in trans? Or, maybe, does it indicate that the estimation procedure itself performs suboptimally?

Author’s response: *In our database, transcriptional regulators were parts of many cassettes, but the overall cassette propensity of studied transcriptional regulators was only 35%. We obtained data on all studied genes from the IMG database, where the COGs were assigned by comparing protein sequences to COG PSSMs from the CDD database using RPS-BLAST. This kind of process might not always be ideal for assigning correct specificity to transcriptional regulators and may lead to grouping of regulators responsible for pathways from different segments of metabolism, despite the initial assignment. This could have, in turn, lead to overestimation of singleton regulators involved in our analysis of carbohydrate metabolism genes. Moreover, transcription factors are often duplicated, forming paralogs with same of different specificity, and hence it is difficult to assign specificity to regulators acting in trans (co-localization combined with analysis of binding motifs is a powerful approach, but requires manual examination of each case). On the other hand, many transcriptional regulators carbohydrate metabolism, indeed, work in trans; so their low cassette propensity is not entirely unexpected.*

## Additional files

Additional file 1: Bacterial genomes. 665 studied bacterial genomes, specified by species and strain name and taxonomy data available from GenBank [19]. (XLS 81 kb)

Additional file 2: Clusters of Ortholous Groups of genes (COGs) used in the study. Genes marked in green were added after fusion case analysis described in the Methods section. (XLS 67 kb)

Additional file 3: Distribution of cassette sizes among functional classes. (JPEG 128 kb)

Additional file 4: Distribution of cassette sizes among bacterial taxa. (JPEG 103 kb)

Additional file 5: Histogram of distribution of COGs from different functional classes over cassette propensity. (JPEG 137 kb)

Additional file 6: Co-localization of functional classes. Co-localization of genes from different functional classes, with *P*-value less than 0.00001; mean co-localization numbers in the random simulation are given in column 4. (XLS 6 kb)

Additional file 7: Co-localization of COGs. Most abundant co-localization cases of genes from different COGs. COG pairs in bold have additionally passed the criteria for statistical significance described in the Methods section. (XLSX 15 kb)

## Abbreviations

COG: Cluster of orthologues groups of genes
IMG: The integrated microbial genomes database
