# Supplementary figures and images for: Sugar Lego: gene composition of bacterial carbohydrate metabolism genomic loci
Source: Biol Direct. 2017 Nov 25;12:28. doi: 10.1186/s13062-017-0200-7 (PMC5702140; doi:10.1186/s13062-017-0200-7)

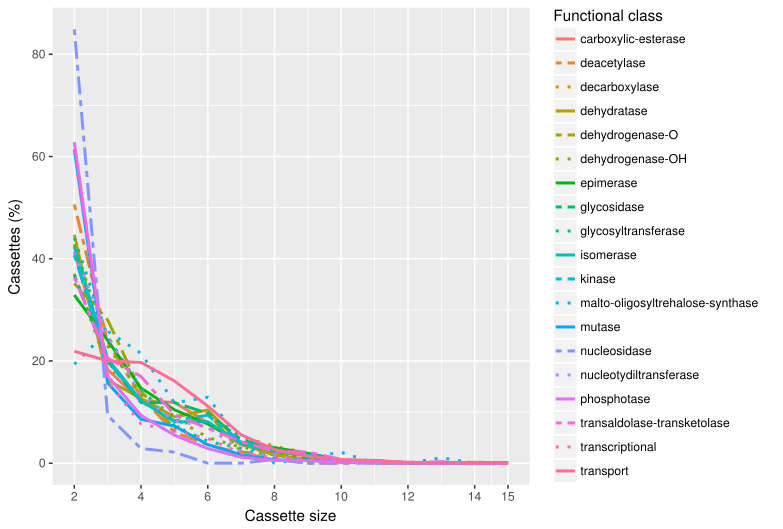

Supplement: Supplementary file 3 — Distribution of cassette sizes among functional classes. (JPEG 128 kb) [file 13062_2017_200_MOESM3_ESM.jpg]

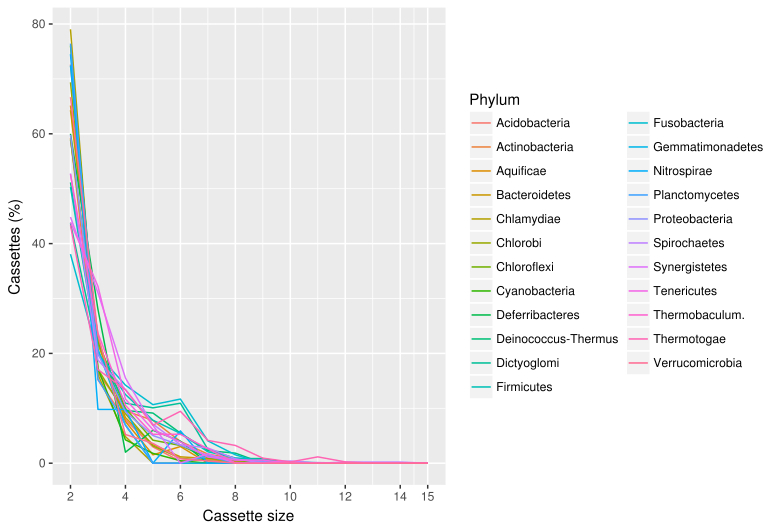

Supplement: Supplementary file 4 — Distribution of cassette sizes among bacterial taxa. (JPEG 103 kb) [file 13062_2017_200_MOESM4_ESM.jpg]

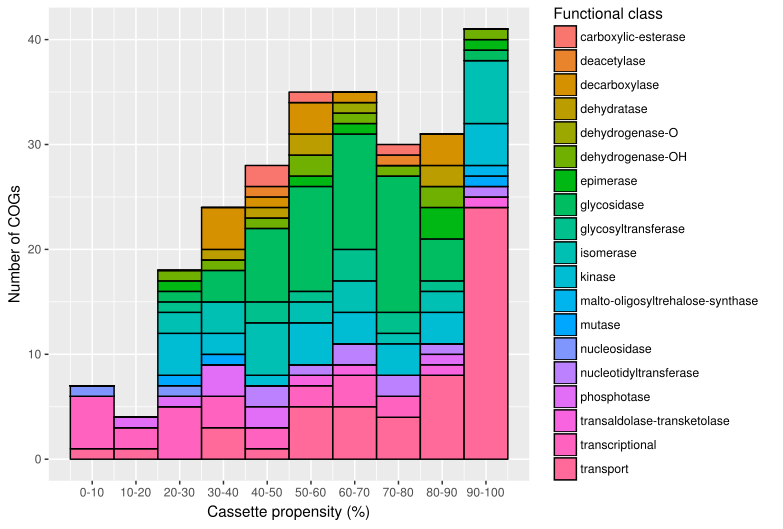

Supplement: Supplementary file 5 — Histogram of distribution of COGs from different functional classes over cassette propensity. (JPEG 137 kb) [file 13062_2017_200_MOESM5_ESM.jpg]
